# Effects of Carbon Nanowalls (CNWs) Substrates on Soft Ionization of Low-Molecular-Weight Organic Compounds in Surface-Assisted Laser Desorption/Ionization Mass Spectrometry (SALDI-MS)

**DOI:** 10.3390/nano11020262

**Published:** 2021-01-20

**Authors:** Ryusei Sakai, Tomonori Ichikawa, Hiroki Kondo, Kenji Ishikawa, Naohiro Shimizu, Takayuki Ohta, Mineo Hiramatsu, Masaru Hori

**Affiliations:** 1 Department of Electronics, Nagoya University, Furo, Chikusa, Nagoya 464-8603, Japan; sakai.ryusei@e.mbox.nagoya-u.ac.jp; 2 Japan Aerospace Exploration Agency, 7-44-1, Jindaiji, Higashi-machi, Chofu-shi, Tokyo 182-8522, Japan; ichikawa.tomonori@jaxa.jp; 3 Center for Low-temperature Plasma Sciences, Nagoya University, Furo, Chikusa, Nagoya 464-8603, Japan; ishikawa@plasma.engg.nagoya-u.ac.jp (K.I.); shimizu@plasma.engg.nagoya-u.ac.jp (N.S.); hori@nuee.nagoya-u.ac.jp (M.H.); 4 Departmet of Electrical and Electronic Engineering, Meijo University, Shiogamaguchi, Tenpaku, Nagoya 468-8502, Japan; tohta@meijo-u.ac.jp (T.O.); mnhrmt@meijo-u.ac.jp (M.H.)

**Keywords:** carbon nanowalls, surface-assisted laser desorption/ionization mass spectrometry, plasma-enhanced chemical vapor deposition

## Abstract

Carbon nanowalls (CNWs), which are vertically oriented multi-layer graphene sheets, were employed in surface-assisted laser desorption/ionization mass spectrometry (SALDI-MS) measurements to detect low-molecular-weight organic compounds. CNWs substrates with widely different wall-to-wall distances from 142 to 467 nm were synthesized using a radical-injection plasma-enhanced chemical vapor deposition (RI-PECVD) system with nanosecond pulse biasing to a sample stage. When survival yield (SY) values of *N*-benzylpyridinium chloride (N-BP-Cl) were examined, which is commonly used to evaluate desorption/ionization efficiency, a narrower wall-to-wall distance presented a higher SY value. The highest SY value of 0.97 was realized at 4 mJ/cm^2^ for the highest-density CNWs with a wall-to-wall distance of 142 nm. The laser desorption/ionization effect of arginine, an amino acid, was also investigated. When CNWs with a narrower wall-to-wall distance were used, the signal-to-noise (SN) ratios of the arginine signals were increased, while the intensity ratios of fragment ions to arginine signals were suppressed. Therefore, the CNWs nanostructures are a powerful tool when used as a SALDI substrate for the highly efficient desorption/ionization of low-molecular-weight biomolecules.

## 1. Introduction

Matrix-assisted laser desorption/ionization mass spectrometry (MALDI-MS) is a powerful method for the rapid, noninvasive, and sensitive sampling of biological fluids due to the soft-ionization properties of biomolecules such as proteins, amino acids, DNA, RNA, as well as synthetic and biopolymers [1,2]. However, it is well known that MALDI mass spectra are generally convolved with peaks of matrix-related fragments, especially in the case of low-molecular-weight analytes with mass-to-charge ratios (*m*/*z*) of less than 700 [2].

To solve this problem, Sunner et al. first reported surface-assisted laser desorption/ionization mass spectrometry (SALDI-MS) as a matrix-free desorption/ionization method in 1995 [3]. SALDI-MS has recently attracted a great deal of attention, especially as a matrix-free method to investigate low-molecular-weight analytes [4,5,6,7]. Various types of SALDI substrates such as Si-based materials (e.g., porous Si [8] and Si nanowires (SiNWs) [9]), carbon-based materials (e.g., carbon nanotubes (CNTs)) [10], graphene [11,12], graphene oxide [12]), and metal-based materials including nanoparticles (e.g., Ge [13], Ag [14], Pt [15], Au [16,17], and TiO_2_ [18]) have been reported. In the case of graphite-SALDI reported in 1995, graphite particles with a size of 2–150 µm were used together with glycerol to efficiently realize the desorption of low-molecular-weight analytes, including proteins and peptides dispersed in glycerol solution [3]. Porous Si with surface roughness on the order of hundreds of nanometers, formed by electric field etching, has also successfully improved sensitivity and suppressed the fragmentation of analytes [8]. It has also been reported that an array of SiNWs with diameters of 10–40 nm, lengths less than 5 µm, and density less than 10 wires/µm^2^ are highly effective for the promotion of soft ionization at lower laser fluence compared with previous cases of porous Si and conventional MALDI [9]. The effect of the surface morphology of the protrusion shapes in SiNWs has been suggested to contribute to the high ionization efficiency [9]. In 2007, Seino et al. conducted a systematic experiment using the SALDI-MS method with germanium nanodots (GeNDs) of different sizes from several tens of nanometers to several hundreds of nanometers, grown on a silicon wafer [13]. As a result, the larger but less-dense GeNDs showed a high signal-to-noise (SN) ratio, and an electric field desorption model of their surface was proposed. According to these results, the surface morphology characteristics of the SALDI substrate, such as the size and shape of the nanostructures as well as the specific surface area, are essential factors to determine desorption and ionization. Furthermore, physicochemical properties such as light absorption, heat capacity, thermal and electrical conductivity, and surface wettability can also affect the analytical performance of SALDI-MS. However, desorption/ionization and fragmentation of the analytes occur simultaneously for multiple reasons, which makes it difficult to determine and optimize the essential factors to realize highly sensitive and selective SALDI, especially for low-molecular-weight analytes such as proteins and peptides. In recent years, *N*-benzylpyridinium chloride (N-BP-Cl) has generally been used as a chemical thermometer to indicate the extent of ionization fragments [19,20,21,22,23,24,25]. The survival yield (SY) of N-BP-Cl is defined using the intensities of benzylpyridinium ions ((BP)^+^) and benzylium ions ((C_7_H_7_)^+^), as described in detail later. SiNWs, which were first applied to SALDI-MS in 2006, showed higher SY than porous Si due to their unique surface morphology [20]. Carbon-based materials such as CNTs and graphite have exhibited high SY values due to the light-absorption characteristics peculiar to carbon-based materials and phase transition/destruction [21]. Pt nanoflowers (PtNFs) were reported to show a high SY of 0.97, even at low laser fluence (about 10 mJ/cm^2^), due to their protrusion shape, which causes concentration of an electric field [22]. It has been shown that ionization with low laser fluence is effective for achieving a high SY. In addition, GaP nanoparticles [23] and metal nanoparticles (Au, Ag, Pt, Pd) [24] exhibit light absorption efficiency, which is also important for soft-ionization ability. The correlation between ion desorption efficiency, internal energy transfer, and surface morphology was recently discussed with respect to control of the surface morphology of Si nanopillars [25]. However, systematic experiments and validation have been limited in terms of elucidating the key factors that result in high SY (less fragmentation).

According to these previous reports and considering the viewpoint of soft-ionization performance, carbon nanowalls (CNWs) are a promising candidate of SALDI substrates because they have ideal nanostructures with high-density graphene edges that exhibit excellent light absorption efficiency. CNWs are self-organized nanomaterials comprised of wall-like aggregations of multi-layer nanographene sheets that stand vertically on substrates such as wafers, glass plates, and metal sheets [26,27]. Therefore, they have a high density of graphene edges at the atomic scale on their top regions. It is suggested that the local electric field concentration is induced by a laser irradiation at those edges of the CNWs, and the graphitic properties of the CNWs mean they effectively absorb the energy of photons and thus serve as an energy-transfer medium. As a result, these characteristics have the potential to enhance the laser desorption/ionization efficiency on CNWs substrate. With a focus on this point, Hori et al. invented SALDI-MS using CNWs and obtained patents in 2009 [28] and 2013 [29]. Experimental results were first reported in 2015 [30]. It was also reported in 2017 that some types of metabolites, such as glucose, melamine, dopamine, and acetaminophen, can be detected using SALDI-MS, and the SY was 0.62 when boron-doped CNWs with hydrophobic surfaces were employed as a SALDI substrate [31]. Furthermore, improvements in the efficiency and sensitivity for the desorption/ionization of analytes by surface hydrophilization of CNWs using atmospheric pressure plasma were reported in 2019 [32]. However, this has been limited to the reporting of the measurement of several molecules on CNWs, whereas the laser desorption/ionization mechanism on CNWs has not yet been sufficiently clarified. For example, it has not been studied how the density of SALDI substrates controlled at the nanoscale, which could be one of important factors in SALDI-MS, has a suppression effect on the ion fragmentations by the SY method.

In this study, the SY method, used to investigate the extent of ionization fragments, was applied to CNWs with widely different wall-to-wall distances, which were realized by a radical-injection plasma-enhanced chemical vapor deposition (RI-PECVD) system with a nanosecond pulse biasing to a sample stage [33]. The effect of the CNWs density, which could be one of the important factors in SALDI-MS, was investigated using CNWs with different densities at the nanoscale. Furthermore, the laser desorption/ionization effect of the amino acid arginine was also investigated using the CNWs as a SALDI substrate because amino acids are generally difficult to ionize.

## 2. Materials and Methods

### 2.1. Preparation of CNWs Substrates

CNWs were grown on Si wafers using a radical-injection plasma-enhanced chemical vapor deposition (RI-PECVD) system (Katagiri engineering Co., Ltd., Kanagawa, Japan) [27]. In this system, the generation of radical species is precisely and independently controlled using two types of plasma sources. One is a surface-wave plasma (SWP) source located at an upper region using a microwave (2.45 GHz) power of 400 W. The other source is a capacitively coupled plasma (CCP) source at a lower region that uses a radio frequency (RF) power of 400 W at 100 MHz. The distance between the CCP upper electrode and the lower sample stage was 30 mm. These two plasma sources were connected through a mesh electrode as an upper electrode for the CCP, and 100 sccm CH_4_ and 50 sccm H_2_ gases were introduced into the CCP and SWP chambers, respectively. The total pressure was kept at 1 Pa. The sample temperature was maintained at 650 °C during the entire deposition process. In this experiment, nanosecond (ns) pulse biases were also applied to the sample stage during growth to synthesize widely isolated CNWs [33]. The direct current (DC) voltage input (V_in_) was changed at 0, 90, and 120 V to operate an inductive energy storage (IES) circuit. The pulse frequency of the IES circuit operation was 50,000 pulses per second (pps), that is, every 20 μs. The deposition time for every growth of CNWs was 4 min 50 s.

### 2.2. SALDI-TOF-MS Measurements

A time-of-flight mass spectrometer system (TOF-MS; Toyama Co., Ltd., Kanagawa, Japan) was used in this experiment [32]. The background pressures of the ionization chamber and the TOF chamber were kept below 10^−7^ Pa. The surfaces on the CNWs/Si sample were irradiated with a fourth harmonic wave with a wavelength of 266 nm from an Nd:YAG laser (repetition rate: 30 Hz, pulse width: 2 ns; Spectra-Physics, Inc., CA, USA, Quanta-Ray Pro 250). The ion signals were detected with a microchannel plate (MCP) in the positive ion mode with an application of a high voltage of 2 kV.

Two types of detection targets were used. One was N-BP-Cl (C_12_H_12_ClN; 204 amu) to examine the degree of fragmentation in the desorption process [19,20,21,22,23,24,25]. N-BP-Cl (C_12_H_12_ClN, Alfa Chemistry, Ronkonkoma, NY, USA, ACM2876133) was dissolved in methanol (FUJI FILM Wako Pure Chemical Corporation, Osaka, Japan, 138-06473), and the concentration was adjusted to be 0.1 mM, referencing the previous paper [21]. Then, 5 µL of the N-BP-Cl solution was dropped onto the CNWs/Si sample and dried in the ambient air. The SY of N-BP-Cl is defined as:

(1) $$\mathrm{SY}=\left(\frac{I_{M}}{I_{M}+I_{F}}\right)$$

where I_M_ and I_F_ are the intensities of benzylpyridinium ions ((BP)^+^) and benzylium ions ((C_7_H_7_)^+^) with *m*/*z* of 170 and 91, respectively [21,24]. For N-BP-Cl detection, the laser fluences were set to be 4, 8, and 12 mJ/cm^2^. The other detection target was arginine (C_6_H_14_N_4_O_2_; 174 amu), a typical amino acid, to demonstrate the effect of different wall-to-wall distances of CNWs on biomolecules. L-Arginine (Sigma-Aldrich Co. LLC, St. Louis, USA, A5006) was dissolved in ultrapure water (Milli-Q), and the concentration was adjusted to be 500 mM, which was set higher than in the previous paper to make it easier to measure [32]. The pH of the arginine solution was 10.43. Then, 5 µL of the aqueous solution was dropped onto the CNWs/Si sample and dried in the ambient air. The laser fluence was 8 mJ/cm^2^ for arginine detection.

## 3. Results and Discussion

### 3.1. CNWs Substrates

**Figure 1:**
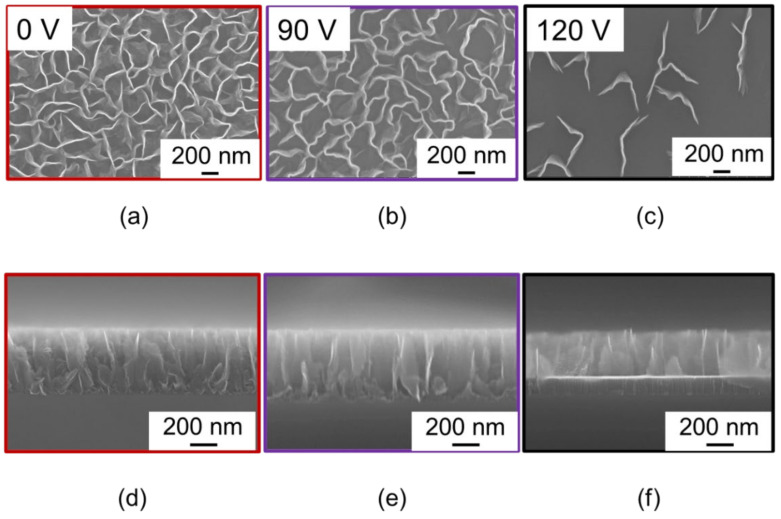
(**a**–**c**) Top-view and (**d**–**f**) cross-sectional-view SEM images of carbon nanowalls (CNWs) synthesized on Si at V_in_ of (**a**,**d**) 0, (**b**,**e**) 90, and (**c**,**f**) 120 V.

Figure 1 shows top- and cross-sectional views of scanning electron microscopy (SEM; Hitachi High Technologies Corporation, Tokyo, Japan, SU8230) images of the CNWs. As shown in the cross-sectional images (Figure 1d–f), the heights of all the CNWs were about 500 nm. Counting the numbers of walls crossing the straight line in the top-view images (Figure 1a–c), the average wall-to-wall distances were defined as their reciprocal. The average wall-to-wall distances of the CNWs samples were 142, 237, and 467 nm for V_in_ of 0, 90, and 120 V, respectively. As reported previously, a 125 nm thick amorphous-carbon (a-C) film was also formed at the CNWs/Si interface with V_in_ of 120 V [33].

**Figure 2:**
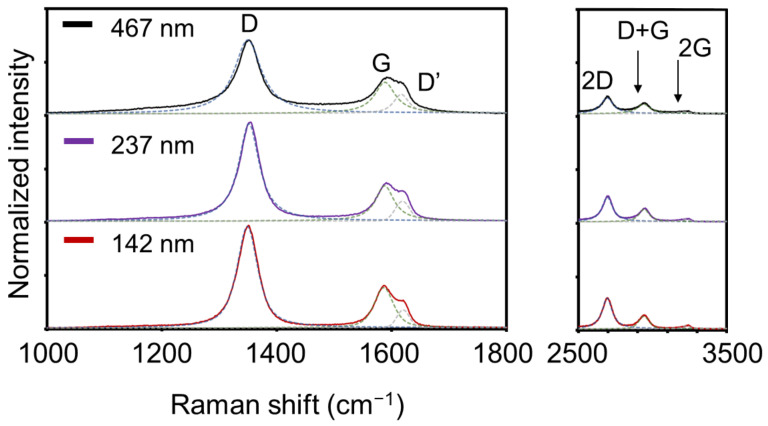
Raman spectra of CNWs with wall-to-wall distances of 142, 237, and 467 nm synthesized at V_in_ of 0, 90, and 120 V, respectively.

**Figure 3:**
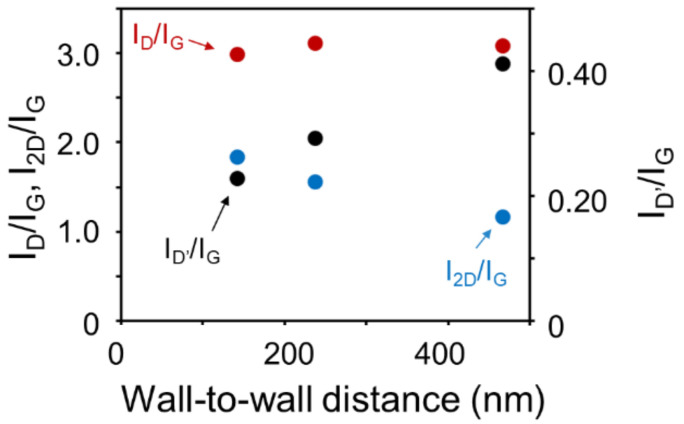
Peak area intensity ratios of I_D_/I_G_, I_D’_/I_G_, and I_2D_/I_G_ in Raman spectra of CNWs with wall-to-wall distances of 142, 237, and 467 nm synthesized at V_in_ of 0, 90, and 120 V, respectively.

Figure 2 shows Raman spectra of the CNWs using laser excitation (Renishaw plc, Wotton-under-Edge, UK, inVia Raman) with a wavelength of 532 nm. Three peaks typically observed in graphene materials were clearly observed in all the spectra. The G-band peaks at 1586 cm^−1^ are attributed to the six-membered ring structure of graphene. The D-band peaks at 1350 cm^−1^ indicate disorder and defects of the six-membered ring structures. The D’-band peaks at 1620 cm^−1^ are also related to the finite size of the graphite crystallites and their edges. The relatively large D- and D’-band peaks are characteristic of CNWs and suggest defects in the six-membered ring structures and graphene edges. In addition, the 2D-band around 2690 cm^−1^, the D + G-band around 2940 cm^−1^, and the 2G-band around 3200 cm^−1^ also indicate graphene-based materials with a large amount of graphene edges. Figure 3 shows area intensity ratios of the D-band to G-band peaks (I_D_/I_G_), those of the D’-band to G-band peaks (I_D’_/I_G_), and those of the 2D-band to G-band peaks (I_2D_/I_G_) as a function of the wall-to-wall distances of the CNWs. The I_D’_/I_G_ values increased with the wall-to-wall distances, while the I_D_/I_G_ values were not significantly changed. However, it is difficult to interpret these trends in intensity change because similar peaks from the underlying a-C layer are superimposed. On the other hand, the I_2D_/I_G_ values decreased as the wall-to-wall distances increased, which indicates a reduction of edge components of the CNWs due to a decrease in the wall density.

The wettability of the CNWs surface was also evaluated by contact angle measurements of water droplets. The CNWs with average wall-to-wall distances of 142, 237 and 467 nm showed average contact angles of 132°, 135° and 132°, respectively, which indicates that all the CNWs samples used in this experiment had relatively hydrophobic surfaces and that their surface wettability was not significantly changed with the wall density.

### 3.2. N-BP-Cl Detection

**Figure 4:**
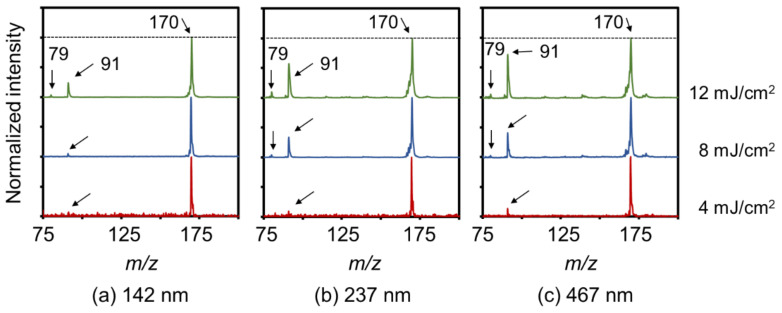
SALDI mass spectra of N-BP-Cl on the CNWs substrates with wall-to-wall distances of (**a**) 142 nm, (**b**) 237 nm, and (**c**) 467 nm, where the fluences of the incident laser light were set at 4, 8, and 12 mJ/cm^2^, respectively. All spectra were normalized with respect to the intensity at *m*/*z* = 170.

**Figure 5:**
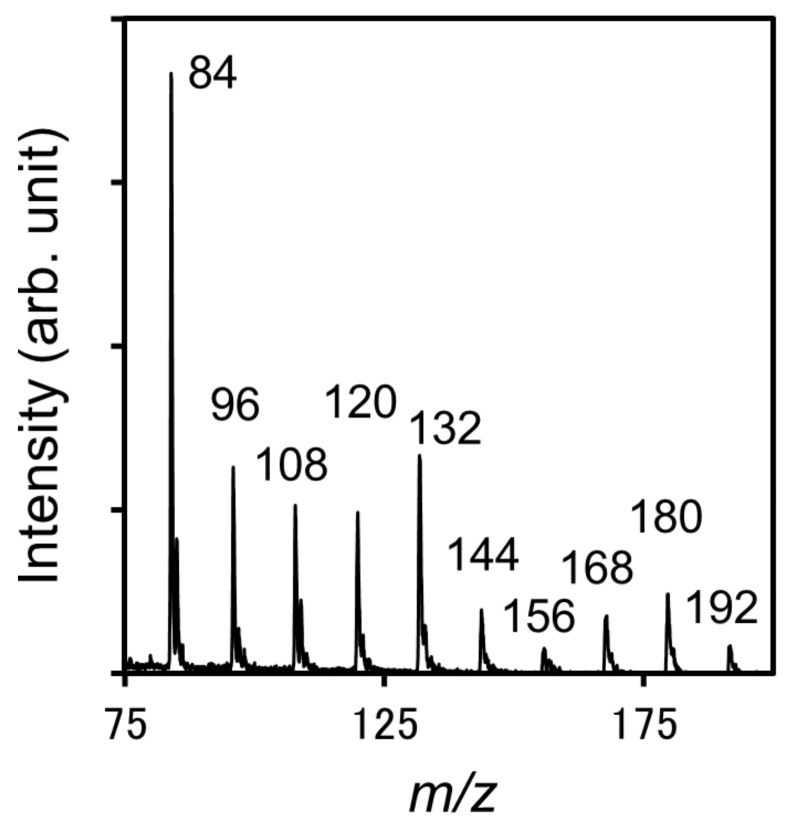
SALDI mass spectrum of CNWs without N-BP-Cl, measured at a laser fluence of 51 mJ/cm^2^.

Figure 4 shows SALDI mass spectra of N-BP-Cl on the CNWs substrates with different wall-to-wall distances. The fluences of the incident laser light were set at 4, 8, and 12 mJ/cm^2^. All spectra are shown normalized with respect to the intensity at *m*/*z* = 170. The spectrum for only the CNWs without N-BP-Cl, measured at a laser fluence of 51 mJ/cm^2^, is shown in Figure 5 for comparison. The two characteristic signals that correspond to (BP)^+^ ions and (C_7_H_7_)^+^ ions were observed at *m*/z = 170 and 91, respectively, regardless of the wall-to-wall distances of CNWs or the laser fluence [21,24]. In addition, relatively small peaks of pyridine ions ((C_5_H_5_N)^+^) also appeared at *m*/*z* = 79 in all the spectra. On the other hand, in the case of only the CNWs without N-BP-Cl, many characteristic peaks were found at *m*/*z* = 84, 96, 108, 120, 132, 144, 156, 168, and 192, as shown in Figure 5. All of these *m/z* values correspond to an integral multiple of 12, which is the atomic weight of carbon; therefore, these signals are presumed to be decomposition products of CNWs. In the measurements of N-BP-Cl on CNWs substrates, the decomposition signals of CNWs were found to be much smaller than the signals of N-BP-Cl at any CNWs wall-to-wall distance and laser fluence. The decomposition signals of CNWs were further suppressed as the wall-to-wall distances of CNWs were narrower or the laser fluence was lower. Therefore, these results indicate that the laser irradiation preferentially excited the desorption/ionization of N-BP-Cl on the CNWs substrates, keeping the decomposition of CNWs sufficiently low.

**Figure 6:**
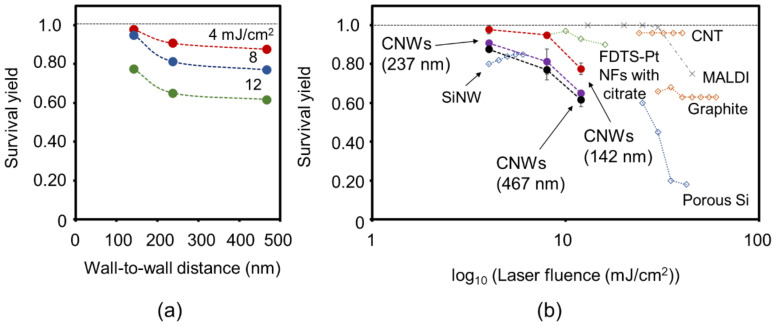
Survival yield (SY) of N-BP-Cl desorbed from substrates with different wall-to-wall distances CNWs as a function of (**a**) wall-to-wall distance and (**b**) laser fluence. (SYs for other nanomaterials were taken from the literature (Si nanowires (SiNWs) and porous Si [20]; perfluorodecyltrichlorosilane-modified platinum nanoflowers (FDTS-PtNFs) with citrate and MALDI (a-cyano-4-hydroxycinnamic acid (CHCA)) [22]; carbon nanotubes (CNTs), graphite [21]).

Figure 6a,b shows SY values of N-BP-Cl on CNWs substrates with different wall densities as a function of the wall-to-wall distance and the laser fluence, respectively. Values reported using other types of nanomaterials as SALDI substrates are also shown in Figure 6b for comparison [20,21,22]. In Figure 6a, the SY values increased as the wall-to-wall distance decreased (higher wall density), regardless of the laser fluence. For example, in the case of the laser fluence of 4 mJ/cm^2^, the average SY values were 0.97, 0.90, and 0.87 with the CNWs substrates, of which the average wall-to-wall distances were 142, 237, and 467 nm, respectively. On the other hand, when the laser fluence was decreased, the SY values became larger (Figure 6b). As a result, the highest SY value of 0.97 was realized at 4 mJ/cm^2^ in the highest-density CNWs sample (wall-to-wall distance of 142 nm). Similar dependence on the laser fluence was also found when using porous Si, PtNFs, and graphite. Regarding the unique morphology and protrusion shapes of SALDI substrates, Luo et al. reported an SY value of about 0.8 on SiNWs at a laser fluence of 4 mJ/cm^2^ [20]. Kawasaki et al. also reported an SY value of 0.97 on perfluorodecyltrichlorosilane-modified platinum nanoflowers (FDTS-PtNFs) with citrate buffer at a laser fluence of about 10 mJ/cm^2^ [22]. Tang et al. also reported an SY value of 0.964 ± 0.023 with CNTs at 25 mJ/cm^2^ [21]. Compared with these reports, the high SY values close to 1 observed in this study were successfully realized at the lowest laser fluence of 4 mJ/cm^2^ when the highest-density CNWs were employed.

As shown in Figure 4 and Figure 6, the ionization and desorption of N-BP-Cl with less fragmentation were realized on the dense CNWs. On the other hand, fragmentation of the target molecules significantly increased with a sparse density of CNWs. These results suggest that one of the dominant factors in SALDI-MS using CNWs is the surface morphology itself rather than the small difference in crystallinity and wettability described in Section 3.1. Furthermore, according to the SEM images shown in Figure 1, it was deduced that the laser desorption/ionization of target molecules occurs at two locations. One is the top edges of the CNWs, which induce efficient desorption/ionization with less fragmentation, that is, the efficient desorption/ionization of target molecules on the edges of CNWs could suppress their fragmentation. The other location is a-C surfaces at the bottom between the walls or sidewalls of the CNWs, where the desorption/ionization of N-BP-Cl are not efficient and larger fragmentation occurs. The CNWs have excellent field-emission characteristics on their top edges; therefore, the local electric-field concentration at the graphene-edges of CNWs can be considered as one of the factors to realize such efficient ionization and desorption [34].

### 3.3. Arginine Detection

**Figure 7:**
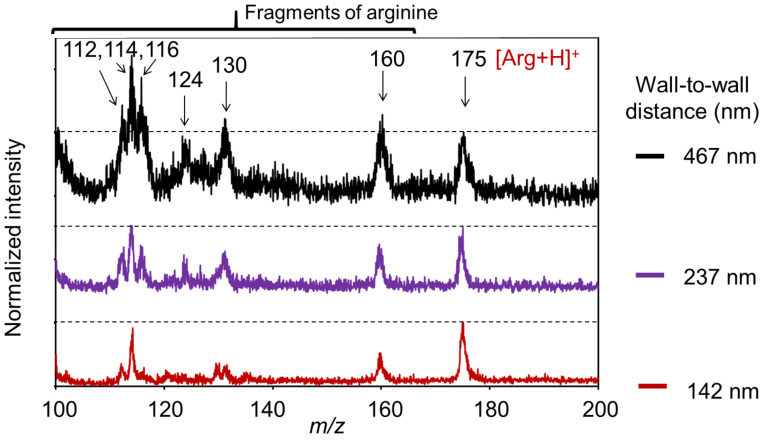
SALDI mass spectra of arginine using CNWs substrates with different wall-to-wall distances (normalized with respect to the intensity of [Arg+H]^+^ at *m*/*z* = 175).

**Figure 8:**
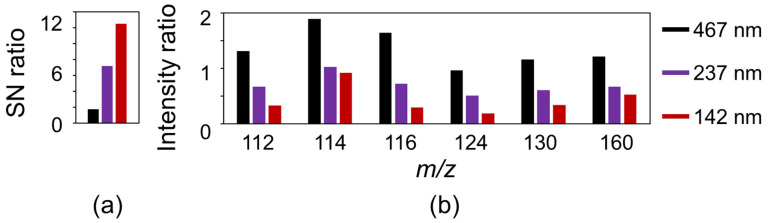
(**a**) Signal-to-noise (SN) ratios of arginine (*m*/*z* = 175) and (**b**) intensity ratios of fragment ions to arginine as a function of the wall-to-wall distance of the CNWs.

Figure 7 shows SALDI mass spectra of arginine using CNWs substrates with different wall-to-wall distances, measured with a laser fluence of 8 mJ/cm^2^. All spectra were normalized with respect to the intensity of arginine [Arg + H]^+^ at an *m/z* value of 175, which are shown as dotted lines. [Arg + H]^+^ signals were clearly observed in all spectra, regardless of the wall-to-wall distances of the CNWs [35]. Signals corresponding to fragment ions of arginine also appeared at 160, 130, 124, 116, 114, and 112 [32]. The fragment ions of arginine, *m*/*z* = 112 and 116, were assigned to [Arg + H-NH_3_-HCOOH]^+^ (or [C_5_H_10_N_3_]^+^) and [Arg + H-CH_5_N_3_]^+^ (or [C_5_H_10_NO_2_]^+^), respectively [32,36]. All of those signals appeared smaller and sharper on the CNWs with narrower wall-to-wall distances. Figure 8 shows SN ratios of the arginine signals at *m*/*z* = 175 and the intensity ratios of fragment ions (*m*/*z* = 112, 114, 116,124, 130, and 160) to arginine (*m*/*z* = 175). As for N-BP-Cl, fragment ions were efficiently suppressed on the denser CNWs in the arginine measurements. In addition, the SN ratios of [Arg + H]^+^ were also significantly improved on the denser CNWs. Thus, arginine, which is a typical biomolecule with low molecular weight (*m*/*z* < 700) and generally difficult to detect, can be clearly detected using the CNWs substrates. By employing CNWs with a narrower wall-to-wall distance, the SN ratios of the arginine signals could be increased, and the intensity ratios of fragment ions to arginine signals could be suppressed. This result indicates that the denser CNWs substrates can more effectively promote the ionization of arginine while suppressing its decomposition. One possible reason for these phenomena is the local electric field concentration on the top edges of the CNWs as described in Section 3.2. Therefore, the CNWs substrates with a high density of graphene edges on their top surface are promising candidates as high-sensitivity SALDI substrates to detect especially low-molecular-weight biomolecules. However, to systematically elucidate their ionization/desorption mechanism on the CNWs substrates, further studies (e.g., evaluating the soft-ionization efficiency of various types of biomolecules and other mixtures) are required. Other factors, such as the pH value of solution and concentration of the of analyte are also essential to evaluate.

## 4. Conclusions

The desorption/ionization mechanisms of organic compounds on CNWs substrates were investigated. CNWs substrates with widely different wall-to-wall distances from 142 nm to 467 nm were synthesized on Si wafers with an RI-PECVD system using nanosecond pulse biasing at the sample stage. The desorption/ionization efficiency of N-BP-Cl on the CNWs substrates was evaluated first. Dense CNWs with an average wall-to-wall distance of 142 nm showed higher SY values (less fragmentation) at all laser fluences compared with sparse CNWs (i.e., those with average wall-to-wall distances of 237 or 467 nm). In addition, the SY values increased as the laser fluence decreased, regardless of the wall density. As a result, the highest SY value of 0.97 was realized at a laser fluence of 4 mJ/cm^2^ in the highest-density CNWs with an average wall-to-wall distance of 142 nm.

Furthermore, the laser desorption/ionization effect of the amino acid arginine was also investigated using CNWs with widely different wall-to-wall distances because amino acids are generally difficult to ionize by conventional MALDI-MS and SALDI-MS, even with many types of nanomaterial substrates. As a result, when the CNWs with a narrower wall-to-wall distance were used, the SN ratios of the arginine signals were increased while the intensity ratios of fragment ions to arginine signals were suppressed.

The results presented here indicate that the nanostructures of the CNWs edges efficiently promote the desorption/ionization of low-molecular-weight organic compounds, while their decomposition is prevented. Therefore, these unique and superior CNWs substrates will open the way to a novel laser-induced desorption/ionization technique for the high-efficiency detection of low-molecular-weight biomolecules.

## Data Availability

The data presented in this study are available on request from the corresponding author.
